# Insurance Stability and Cancer Screening Behaviors

**DOI:** 10.1089/heq.2018.0093

**Published:** 2019-04-03

**Authors:** Karen M. Freund, Sarah A. Reisinger, Amy M. LeClair, Grace H. Yoon, Sarah M. Al-Najar, Gregory S. Young, Evelyn T. González, Jill M. Oliveri, Electra D. Paskett

**Affiliations:** ^1^ Institute of Clinical Research and Health Policy Studies, Department of Medicine, Tufts Medical Center, Tufts University School of Medicine, Boston, Massachusetts.; ^2^ Department of Population Sciences, Comprehensive Cancer Center, Ohio State University, Columbus, Ohio.; ^3^ Institute of Clinical Research and Health Policy Studies, Department of Medicine, Tufts Medical Center, Boston, Massachusetts.; ^4^ Tufts University School of Medicine, Boston, Massachusetts.; ^5^ Center for Biostatistics, Department of Biomedical Informatics, Ohio State University, Columbus, Ohio.; ^6^ Fox Chase Cancer Center, Office of Community Outreach, Philadelphia, Pennsylvania.; ^7^ Department of Internal Medicine, Comprehensive Cancer Center, Ohio State University, Columbus, Ohio.

**Keywords:** cancer screening, health disparities, insurance, racial/ethnic minorities

## Abstract

**Background:** Disparities in rates of cancer screening are observed in underserved populations. Lack of stable health insurance may contribute to these disparities. The goal of this study was to examine the association between insurance stability and up-to-date cancer screening in underserved populations.

**Methods and Findings:** We enrolled 333 community participants aged 40–74 years across four different sites in three states: Chinese Americans in Boston, Massachusetts; Hispanics in Columbus, Ohio; Appalachian populations from Ohio's Appalachian counties; and Blacks and African Americans in Philadelphia, Pennsylvania. Self-reported screening rates were 77.9% for breast cancer, 71.1% for cervical cancer, and 67.7% for colorectal cancer (CRC). Screening rates fell short of Health People 2020 targets for breast, colorectal, and cervical cancer screenings. Being currently insured was associated with current CRC screenings (69.7% among insured vs. 30.7% among uninsured, *p*=0.0055), but not with breast or cervical cancer screenings. Stable 12-month insurance coverage was not statistically associated with up-to-date screenings.

**Conclusion:** Having current insurance was associated with CRC screening; stability of insurance was not associated with cancer screening. Insurance coverage alone is not the main driver of cancer screening.

## Introduction

Health disparities are prevalent among immigrant, low-income, low-educated and ethnic minority populations. Of the many disparities they face, minorities have markedly lower rates of cancer screening than their non-Hispanic White, English-speaking counterparts.^1^ Lack of health insurance coverage^2^ and being non-White have been attributed to lower screening rates and later cancer detection.^3^ For example, while 65% of all Americans between ages 50 and 75 years are up-to-date with colorectal cancer (CRC) screening, only 53% of Hispanics and 37% of uninsured individuals are up-to-date.^4^

The direct effect of lack of insurance coverage on cancer screening among multiple racial and ethnic minority groups is well documented. Lower adherence to annual Papanicolaou (Pap) tests is associated with being Hispanic and uninsured.^5^ Among Asian American women, being privately insured and having a usual source of care are strong predictors of receiving both breast and cervical cancer screening tests.^6^ Among those without insurance, recent immigrants are less likely to have Pap tests than U.S. born counterparts.^7^ In urban settings, lower breast cancer screening rates are attributed to being uninsured and residing in low-income communities without local facilities for preventive care.^8^ Appalachia is a region of the United States, which experiences significant disparities in cancer incidence and mortality compared with the rest of the country, attributed to high rates of poverty and low rates of educational attainment, primary care access, and insurance.^9^ Within rural Appalachian counties, 18.2% of people younger than 65 years are uninsured, compared with 16.8% in the nation overall. Nationally, women in states that are not participating in Medicaid expansion had lower odds of receiving annual mammograms and Pap tests, with larger differences among the uninsured population.^10^

Although the positive impact of insurance coverage on cancer screening in minorities is well established, there is still a gap in research on the impact of stable health insurance on cancer screening behaviors in racial and ethnic minorities. The aim of this study was to examine the association between current insurance status, insurance stability, and cancer screening among participants from four different underserved populations in three states.

## Methods

### Study design and patient population

As part of the Cancer Disparities Research Network,^11^ a regional entity of the National Cancer Institute's Geographic Management of Cancer Health Disparities Program, we conducted a cross-sectional analysis to investigate the association between current insurance status and insurance stability with breast, cervical, and CRC screening rates. Participants were recruited across four different sites in three states: Chinese Americans were recruited in Boston, Massachusetts; Hispanic populations in Columbus, Ohio; Appalachian populations from Ohio's Appalachian region; and African American and Black populations in Philadelphia, Pennsylvania. Subjects were recruited from community populations, including community-based organizations, faith-based organizations, public housing, screening events, health fairs, and contacting subjects from existing research studies. No recruitment was conducted in clinical settings.

Participants were eligible if they were aged between 40 and 74 years and lived in an Appalachian county or self-identified as African American, Hispanic, or Asian. Participants also had to meet at least one of the following underserved criteria: live in a medically underserved area (as defined by Health Resources and Services Administration),^12^ have low literacy (defined as low proficiency in English or, for English speakers, a score of six or less on the Rapid Estimate of Adult Literacy in Medicine Short Form^13^ or three or greater on the Single Item Literacy Screener),^14^ have a household income lower than 100% of the federal poverty level in 2015 ($11,670 for a single individual or an annual income of $23,850 for a family of four), or be uninsured or receive subsidized health insurance coverage (Medicaid or subsidies on the insurance exchange). Exclusion criteria included those who lived in a nursing home or other institution, were pregnant, or had a prior invasive cancer diagnosis.

All participants in the study completed the informed consent process. They were compensated with a $10 gift card for participating in the baseline survey. Surveys were available in English, Spanish, or Chinese, and self-administered on a tablet or computer or verbally administered by project staff. The study was approved by the institutional review boards at the Ohio State University, Tufts Medical Center, and Fox Chase Cancer Center.

### Measures

Participants answered questions about their age, race/ethnicity, income, highest level of education, and primary care physician (PCP) status. We collapsed age into three categories: 40–50, 51–64, and 65–74 years. We created four race/ethnicity categories: Hispanic, non-Hispanic White, non-Hispanic Black, and Chinese. Annual income was collapsed into four categories: less than $15,000, $15,000–$24,999, $25,000–$49,999, and $50,000 or more. We collapsed education into three categories: some high school or less, some college or less, or obtained a college degree or more.

### Insurance measures

We categorized insurance coverage into the following categories: private, Medicare, Medicaid, other including subsidized coverage, and uninsured. Participants were asked about their current insurance status, as well as the number of switches and/or gaps in insurance coverage in the previous 12 months. We defined current insurance status as whether or not the participant was actively insured at the time of the baseline survey. We defined unstable insurance coverage as being uninsured, losing coverage, or changing among the defined insurance categories at any point in the 12 months before the survey.

### Up-to-date cancer screening

We asked participants about their recent breast, cervical, and CRC screenings. Our definition of up-to-date cancer screening is based on the United States Preventive Services Task Force (USPSTF) guidelines. Female participants aged 40–74 years were considered up-to-date with breast cancer screening if they had received a mammogram in the last 2 years.^15^ Female participants aged 40–65 years were considered up-to-date with cervical cancer screening if they received a Pap test in the last 3 years or a Pap test and human papillomavirus (HPV) test in the last 5 years.^16^ Participants aged 50–74 years were considered up-to-date on CRC screening if they received a fecal occult blood test in the last year, a flexible sigmoidoscopy in the last 5 years, or a colonoscopy in the last 10 years.^17^ Table 1 summarizes the screening guidelines and lists the Healthy People 2020 goals for population levels of each screening.

**Table 1.** Definitions of Up-to-Date Cancer Screening and Healthy People 2020 Target Screening Rates for Breast, Cervical, and Colorectal Cancer

USPSTF guidelines	Population	Screening examination	Healthy people 2020 target
Breast cancer	Women aged 40–74 years	Mammogram in last 2 years	81.1%
Cervical cancer	Women aged 40–65 years	Pap test in last 3 years	93.0%
		Pap and HPV test in last 5 years	
CRC	Men and women aged 50–74 years	FOBT/FIT in last year	70.5%
		Flexible sigmoidoscopy in last 5 years	
		Colonoscopy in last 10 years	

CRC, colorectal cancer; FIT, fecal immunochemical test; FOBT, fecal occult blood test; HPV, human papillomavirus; Pap, Papanicolaou; USPSTF, United States Preventive Services Task Force.

### Analysis

We performed descriptive analyses, looking for associations between health insurance status and health insurance stability with demographic characteristics, including age, race/ethnicity, income, and highest level of education, using Fisher's exact test. We analyzed the association between up-to-date breast, cervical, and CRC screenings and the same demographic variables. We tested the association between individual-level demographic factors and up-to-date screening status, and between up-to-date screenings and our two insurance variables (i.e., insurance status and insurance stability).

## Results

Of 333 total participants across all three clinical sites, 30.0% were recruited from Boston, Massachusetts; 30.9% were from Philadelphia, Pennsylvania; and 39.0% were from Columbus, Ohio; and Appalachian counties in Ohio. Table 2 shows participant demographics by current insurance status and previous 12-month insurance stability. Women comprised 65.7% of the study cohort. A majority (85.8%) of participants were older than 50 years, 8.4% identified as Hispanic, 29.8% as non-Hispanic White from Appalachia, 31.6% as non-Hispanic Black, and 30.1% as Chinese. English was the proficient language for 62.1% of the participants, whereas 29.8% reported Chinese and 8.1% reported Spanish as their primary language. The cohort reported low income, with 62.2% reporting an annual household income of less than $25,000. Rates of insurance coverage were high, with 90.7% reporting being currently insured at the time of the interview and 69.9% reporting stable insurance coverage over the past 12 months.

**Table 2.** Demographic Characteristics and Insurance Status—Cancer Disparities Research Network

Variable	Level	*N*	Currently insured	Fisher's *p*-value	Insurance stability	Fisher's *p*-value
Gender	Male	114	95.6%	0.0282	76.3%	0.0777
	Female	218	88.1%		66.5%	
Age, years	40–50	47	61.7%	<0.0001	36.2%	<0.0001
	51–64	198	93.9%		74.2%	
	65–74	87	98.9%		78.2%	
Race/ethnicity	Hispanic	28	28.6%	<0.0001	14.3%	<0.0001
	Non-Hispanic White	99	99.0%		82.8%	
	Non-Hispanic Black	105	94.3%		67.6%	
	Chinese	100	96.0%		75.0%	
Preferred language	English	206	96.1%	<0.0001	74.8%	<0.0001
	Spanish	27	29.6%		14.8%	
	Chinese	99	96.0%		74.7%	
Annual household income	Less than $15,000	111	91.0%	0.1496	67.6%	0.6534
	$15,000–$24,999	86	88.4%		68.6%	
	$25,000–$49,999	64	89.1%		68.8%	
	$50,000 or more	56	98.2%		76.8%	
Education	Some high school or less	74	86.5%	0.3207	63.5%	0.4016
	Some college or less	169	92.3%		71.0%	
	College degree+	84	90.5%		72.6%	
Has regular primary care provider	No	24	75.0%	0.0172	45.8%	0.0110
	Yes	304	91.8%		71.7%	

### Demographic information

Current insurance status and insurance stability were associated with age, race/ethnicity, preferred language, and PCP status (Table 2). Participants in the youngest age group, 40–50 years old, were less likely to be insured at the time of the baseline survey compared with those aged 51–64 years and those aged 65–74 years (62% vs. 94% vs. 99%, *p*≤0.0001). Participants aged 40–50 years were less likely to have had stable insurance during the prior year compared with those aged 51–64 or 65–74 years (36% vs. 74% vs. 78%, *p*≤0.0001). Non-Hispanic Whites were most likely, whereas Hispanics were least likely, to be currently insured (99% vs. 29%, *p*≤0.0001) or stably insured (82% vs. 14%, *p*≤0.0001). Participants who reported having a stable PCP were more likely to be currently insured than those who did not have a stable PCP (92% vs. 75%, *p*=0.0172). Those with stable PCPs were also more likely to be continuously insured than those without stable PCPs (72% vs. 46%, *p*=0.0110). There was no significant difference in either insurance status or insurance coverage stability based on gender, income, education, or state of residence.

### Insurance stability and screening rates

We examined whether insurance status and stability were associated with participants’ screening rates (Table 3). Current insurance status was significantly associated with rates of CRC screening (69% among those insured vs. 31% among those uninsured, *p*=0.0055). Insurance stability was not associated with CRC screening. Neither insurance status nor stability was associated with up-to-date breast or cervical cancer screening rates.

**Table 3.** Rates of Up-to-Date Cancer Screening by Insurance Status and Insurance Stability—Cancer Disparities Research Network

Insurance Status	*N*	Breast cancer screening rates	Fisher's *p*-value	*N*	Cervical cancer screening rates	Fisher's *p*-value	*N*	CRC screening rates	Fisher's *p*-value
Currently insured	192	79.2%	0.3114	145	69.7%	0.3471	271	69.7%	0.0055
Currently uninsured	26	69.2%		25	80.0%		13	30.7%	

Insurance Stability	*N*	Breast cancer screening rates	Fisher's *p*-value	*N*	Cervical cancer screening rates	Fisher's *p*-value	*N*	CRC screening rates	Fisher's *p*-value
Stable insurance	145	80.6%	0.2250	109	73.4%	0.4804	214	70.1%	0.1867
Unstable insurance^a^	73	72.6%		61	67.2%		70	61.4%	

^a^ Unstable insurance coverage is defined as being uninsured, losing coverage, or changing among the defined insurance categories within the past 12 months.

## Discussion

Among a cohort of underserved adults, as defined by minority race/ethnicity or low income and education, who lived in Medicaid expansion states, overall self-reported screening rates were 78% for breast cancer, 71.2% for cervical cancer, and 67.7% for CRC. A greater percentage of participants were up-to-date on breast and cervical screenings compared with CRC screenings, regardless of insurance status or stability. The study cohort did not meet any of the Healthy People 2020 initiative target goals for breast, cervical, and CRC screenings.^18–20^ We found an association between insurance status and CRC screening. Participants who were insured were more likely to be up-to-date on CRC screening than those who were not insured. Current insurance status was not associated with being up-to-date on breast or cervical cancer screenings, and we found no significant associations between insurance coverage stability and up-to-date screenings.

Our study is consistent with the findings of other studies regarding insurance status and cancer screening. For CRC screening in particular, prior research reported that uninsured participants were less likely to have ever been screened or up-to-date on screening.^21^ Other studies have similarly found that uninsured groups consistently had lower rates of CRC screening; in fact, the uninsured were less likely to have ever completed any testing, or be up-to-date on CRC screenings, compared with their insured counterparts.^22,23^ One explanation for lower CRC screenings might be that they usually happen by physician referral, and uninsured participants without access to a PCP are not likely to receive recommendations for screenings. Studies have found that there is an association between physician recommendation and CRC screening behaviors—a recommendation can produce a dramatic increase in screening.^23,24^

Of the three cancer screenings evaluated in this study, participants had the lowest screening rates for CRC. This finding might be due to the low availability of free or low-cost colon screening programs. CRC screenings, specifically colonoscopies, are more expensive due to costs of procedure time and anesthesia, and they are often more intensive, requiring much longer preparation time and detailed preparation instructions from a provider prior the visit. Breast and cervical screenings are more easily accessible for the under- and uninsured due to the Centers for Disease Control and Prevention National Breast and Cervical Cancer Early Detection Program (NBCCEDP 2017).^25^ Implementation of NBCCDEP might also explain why our study did not find an association between breast or cervical cancer screenings and insurance status and insurance stability; women were still able to get screened, regardless of insurance.

The study had several limitations. The sample gathered was not population-based, and different recruitment methods were utilized at each site. Although not population-based, all sites recruited from community settings and not clinical practices where patients would be more likely to receive cancer screening than the community population overall. Some of the screening tests, such as colonoscopy within 10 years, may have occurred before the insurance exposure. The Hispanic subsample was much smaller than the other groups, was younger, and more likely to be uninsured; therefore, the ability to measure differences between racial and ethnic groups may have been limited. All the cancer screening data are self-reported.

Despite being a low-income cohort, the majority of our participants had insurance coverage, perhaps reflecting Medicaid expansion post-Affordable Care Act implementation. However, our cohort failed to meet Healthy People 2020 guidelines for three of the most common types of cancer screenings. Stable and current insurance alone do not result in screening; interventions to address access, patient preferences, and motivation are also necessary to meet screening targets.

## Abbreviations Used

CRC: colorectal cancer
FIT: fecal immunochemical test
FOBT: fecal occult blood test
HPV: human papillomavirus
NBCCEDP: National Breast and Cervical Cancer Early Detection Program
Pap: Papanicolaou
PCP: primary care physician
USPSTF: United States Preventive Services Task Force
